# Professional Responsiveness to Health Literacy: A Scoping Review

**DOI:** 10.3928/24748307-20220418-02

**Published:** 2022-04

**Authors:** Flaviane Cristina Rocha Cesar, Katarinne Lima Moraes, Virgínia Visconde Brasil, Angela Gilda Alves, Maria Alves Barbosa, Lizete Malagoni de Almeida Cavalcante Oliveira

## Abstract

**Background:**

Difficulty in understanding and using health information can harm the patient and increase the cost of care provided. So, this study classified and mapped the characteristics and interventions that make health care professionals responsive to the patient's health literacy.

**Methods:**

Medline (PubMed), CINAHL (EBSCO), PsycInfo, ERIC (ProQuest), Lilacs (BVS) and EMBASE (Elsevier) were searched using a combination of controlled descriptors. The selected studies needed to address the concept or main focus of the study among health care professionals in the care or academic environment.

**Key Results:**

After reviewing 34 articles, 14 definitions and 10 subcategories of responsiveness were identified, and a broad characterization of health professional responsiveness to health literacy was proposed. Professional responsiveness to health literacy was characterized as knowing the definition and implications of health literacy for the patient's well-being and being able to develop, adapt, implement, and evaluate health education strategies. Nineteen strategies were mapped for education to ensure professional responsiveness to health literacy, classified as (A) expository (*n* = 18; 94.7%), (B) interactive (*n* = 9; 47.4%), (C) practice with educational materials (*n* = 2; 10.5%), (D) practice with standardized patient or simulation (*n* = 8; 42.1%), and (E) practice with actual patients (*n* = 4; 21.1%).

**Discussion:**

These characteristics and interventions provide a useful taxonomy for the development of curricula and professional education programs, and for the validation and use of measures to evaluate the health workforce. [***HLRP: Health Literacy Research and Practice*. 2022;6(2):e96–e103.**]

**Plain Language Summary:**

We found 14 definitions and 10 categories of professional responsiveness to health literacy. Professional responsiveness to health literacy was characterized as knowing the definition and implications of health literacy for the patient's well-being and being able to develop, adapt, implement, and evaluate health education strategies. Nineteen strategies were mapped for education to ensure professional responsiveness to health literacy.

Health literacy (HL) involves people's “knowledge, motivation, and skills to access, understand, evaluate, and implement health information to make life-long health care decisions” (Sørensen et al., 2012, p. 3). People with low health literacy have higher medical costs and are less efficient when using services than people with adequate health literacy (Palumbo, 2017).

Possible health outcomes of HL include adherence to medication treatment, access to care, lower risk of disease exacerbation, decreased preventable hospitalization rates (Palumbo, 2017), reduced excessive use of health services (Zhang et al., 2019), improved quality of life (Zheng et al., 2018) and reduced length of hospital stay (Wright et al., 2018). Therefore, being responsive to patient HL is a social, economic, and effective health service concern.

A recent study showed that less than 10% of patients stated that they understood oral or written information provided by health care professionals (HCPs) (Rafferty et al., 2020). This data reinforce the need for health services to include HL in their practices; the assessment of indicators of its implementation in the care is the first step in establishing a baseline of the processes being undertaken (Innis et al., 2017; Kripalani et al., 2014).

Preparing the workforce is seen as an attribute for an organization to be literate or responsive to HL (Brach et al., 2012). Limited knowledge of HCPs about HL can negatively affect the patient's well-being and compromise response from the health care services to their needs (Güner & Ekmekci, 2019).

Studies show that nursing students have moderate participation in HL-related learning activities (Maduramente et al., 2019); in addition, one-half of the physicians and nurses studied had never heard about HL during their formal education (Güner & Ekmekci, 2019). In addition, medical residents who evaluated patients' HL level either under- or overestimated their abilities (Zawilinski et al., 2019). On the other hand, physicians, pharmacists, and nurses who had contact with the term or concept of HL showed more positive attitudes, such as using communication strategies focused on HL, and implementing HL programs (Rajah et al., 2017).

In the context of literacy responsive organizations, most studies focused on patient-related interventions and outcomes (Zanobini et al., 2020). Although some studies identified professional competencies in health literacy, they were restricted to the development of teaching plans or pedagogical objectives (Chang, Chen, Liao, et al., 2017; Chang, Chen, Wu, et al., 2017; Coleman et al., 2013). Therefore, it is essential to know which characteristics and interventions are related to the HCPs' responsiveness to patient literacy.

## Professional Competencies and Health Literacy Responsiveness

Competencies presuppose learning as a continuous process of accumulating experiences. This perspective is used to improve, provide, and measure instruction; categorize work responsibilities; and assess individual and organizational capabilities (Miner et al., 2005).

Previous research identified knowledge, skills, and attitudes as HCP competencies in health literacy (Chang, Chen, Wu, et al., 2017; Coleman et al., 2013). Knowledge is related to cognitive processes that enable people to know what to do, how to do it, and when to do it. Skills represent process-oriented knowledge, where the resolution of a given problem or situation is achieved by automatic or normal actions. Professional attitudes are related to values, principles, emotional and relational resources, and reflect one's professional identity (Perrenoud, 2013).

Competencies are a set of schemes based on learning experiences that involve the mobilization and synergism of internal and external resources, resulting in appropriate actions for a given situation. Competence is, therefore, a predictive action (Perrenoud, 2013); consequently, development of knowledge, skills, and attitudes enables HCPs to act more effectively in the presence of a patient with low HL.

In addition, current lists of professional competencies related to HL do not include conceptual agreement (Chang, Chen, Wu, et al., 2017; Coleman et al., 2013), and do not quantify the HL responsiveness in a universal manner. There is also a limitation of interventions in academic curricula, where professionals in clinical practice have not considered much research (Saunders et al., 2018). Thus, this study classified and mapped characteristics of HCPs and interventions that improve their ability to respond to patients' health literacy.

## Materials and Methods

The purpose of a scoping review is to map the existing scientific evidence on a given subject and identify characteristics or factors related to a concept (Munn et al., 2018). It was conducted according to the Joanna Briggs Institute methodology (Peters et al., 2015) and includes review of the issue, inclusion criteria, types of participants, context of the review, concept or main focus, search strategy, data extraction, and presentation of results. The methodology is detailed in the protocol available in Appendix 1 (see https://figshare.com/s/161960aa6503ee329208).

### Research Questions

The research questions of the study are as follows: What are the characteristics of HL responsiveness of HCPs in the care environment? What interventions have been implemented to improve the HL responsiveness of HCPs?

The study used the broad population, concept, and context framework recommended by the Joanna Briggs Institute for Scoping Reviews (Peters et al., 2015). The questions in this review were based on the Joanna Briggs Institute PCC (population-concept-context) model: P = healthcare professionals; C = Characteristics of HL responsiveness of HCPs; C = care or academic environment.

### Search Strategy

The search was conducted in Medline (PubMed), CINAHL (EBSCO), PsycInfo, ERIC (ProQuest), Lilacs (BVS), and EMBASE (Elsevier) databases, using a combination of controlled descriptors (Appendix 1; see https://figshare.com/s/161960aa6503ee329208). Additionally, a search of references of the articles included in the review was performed, selecting publications that met the inclusion criteria of the study. The last search was conducted on April 17, 2020. All searches were combined in Endnote^®^, and duplicate files were removed.

### Inclusion and Exclusion Criteria

Articles published in English, Spanish, and Portuguese were included, most of the studies are in those languages so there was an initial segmentation of scoping in these languages without using additional filters. The selected studies needed to address the concept or main focus of the study among HCPs in the care or academic environment. Studies were excluded that were limited to the evaluation of HL practices without involving intervention; used terms that were correlated but distinct from HL; only addressed project organization; developed a literature review on interventions or education; had materials focused only on the patient; had materials that did not address HL responsiveness; addressed education of other professionals (such as caregivers).

### Main Concept or Focus

The selection of the papers to be read in their entirety was made by checking the adequacy of relationship to the concept or focus of the review, in the title and/or abstract, observing the characteristics of HL- responsiveness of HCPs:

- Knowledge: studies that aimed to assess or describe predominantly cognitive aspects of HCPs on HL.
- Skills: studies that aimed to assess or describe actions of professionals considering the patient's HL, in actual use (clinical practice) or mental aspects (activities that stimulate critical thinking).
- Attitudes: studies that aimed to assess or describe preferences, values, and attitudes of HCPs regarding the HL of patients.

Interventions to improve HL responsiveness were also observed, including studies that aimed to describe the education of HCPs on HL in the care or academic environment.

### Data Extraction and Presentation of Results

A data extraction form has been developed to aid the collection and sorting of key pieces of information from the selected articles: (1) author(s), (2) year of publication, (3) source/country of origin, (4) study population, (5) characteristics of HCPs responsive to HL, and (6) interventions to improve HL responsiveness.

Two levels of analysis were performed for this study. The first level of analysis included the identification of frequent definition or expressions (codes) in the research-collecting instrument. The first author (F.C.R.C.) extracted data from the articles and generated a synthesis of characteristics and interventions, which was later verified by three other authors (K.L.M., V.V.B., L.M.A.C.O.).

In the second level of analysis, the codes were approximated by relationship and convergence. The first author (F.C.R.C.) established the categories of the study, and three other authors (K.L.M., V.V.B., L.M.A.C.O.) analyzed the selected texts for confirmation. In this process, the agreement required for inclusion of categories and subcategories were established at 90% agreement among the researchers.

## Results

### Characteristics of Selected Studies

**Figure 1.**
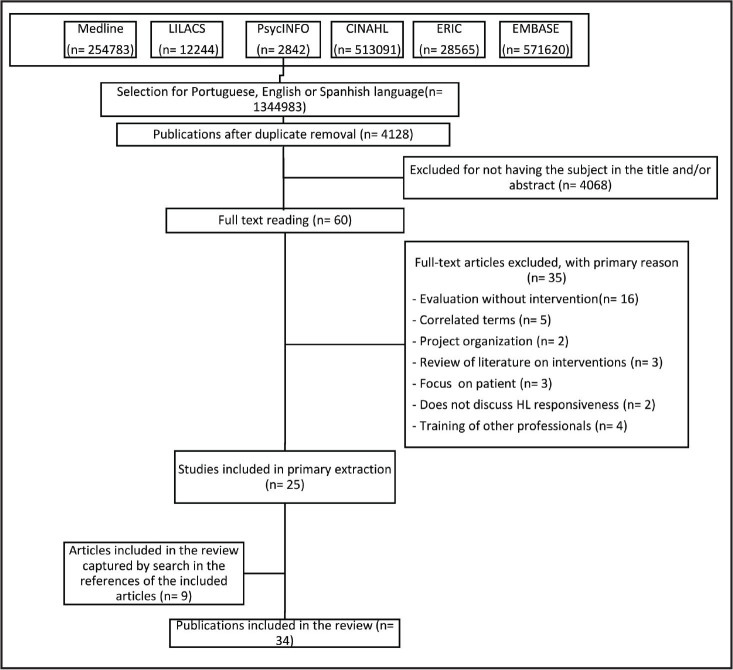
PRISMA flow diagram on health professional responsiveness to health literacy.

The studies included in the final sample (*n* = 34; 100%) were published between 2006 and 2020, all in English (**Figure 1**). Flow diagram for the scoping review process adapted from the PRISMA Extension for Scoping Reviews by Tricco et al. (2018)

Most studies were from the United States (*n* = 24;70.6%), and the others were from Australia (*n* = 4;11.8%), Italy, the Netherlands, Northern Ireland (*n* = 4;11.8%), Turkey (*n* = 1; 2.9%), and China (*n* = 1; 2.9%). The HCPs education regarding HL was provided in more than one-half of the articles (*n* = 19; 55.9%), followed by the characteristics of HL responsiveness of HCPs (*n* = 15; 44.1%). Education that occurred within an academic environment was identified in one-third (*n* = 11; 32.4%) of the studies, one-fifth (*n* = 7; 20.6%) in occurred in the care environment, and in one article (5.3%) it occurred in both locations.

### Characteristics of HL Responsiveness of HCPs in the Care Environment

Fourteen definitions of professional responsiveness to HL were identified. From these, the codes identified in the first level of analysis identified 10 subcategories. The 15 studies included in the subcategories are described in **Table A**.

1. Recognizes definition and effect of HL (*n* = 6; 40%). This subcategory is related to the theoretical knowledge of HCPs, including understanding, delimiting the term (Galati et al., 2018) and recognizing how the HL level affects patient diagnosis and treatment (Güner & Ekmekci, 2019).
2. Recognizes the need to adapt the learning plan and materials to the patient's HL level (*n* = 3; 20%). The use of customized materials was included (Chang, Chen, Wu, et al., 2017), and adaptations of activities were designed to meet the specificities of clients (Coleman et al., 2013).
3. Identifies low HL signs (*n* = 5; 33.3%). This subcategory included mechanisms to assess the patient's HL level, such as use of verbal resources (Galati et al., 2018), body language, and skill to follow instructions (Quinn et al., 2019).
4. Prepares strategies for patient health education (*n* = 8; 53.3%). This included the development of a teaching plan for patients with low HL (Chang, Chen, Wu, et al., 2017) and presentation of information to improve people's understanding of health (Coleman et al., 2008).
5. Implement patient health education strategies (*n* = 4; 26.7%). It included teaching actions (Chang, Chen, Wu, et al., 2017) and patient education (Minnesota Health Literacy Partnership, 2016).
6. Evaluates teaching/intervention strategies (*n* = 4; 26.7%). This described actions to monitor the effectiveness of educational actions (e.g., when the professional confirms understanding of the patients) (Brach et al., 2012).
7. Develops communication focused on health literacy (*n* = 9; 60%). This is related to optimization of communication with simplified explanations (Quinn et al., 2019), and the use of illustrations and examples (Allenbaugh et al., 2019).
8. Develops learning reinforcement strategy (*n* = 3; 20%). This involved stimulating the patient to ask questions (Chang, Chen, Wu, et al., 2017) and summarizing important points on the teaching theme (Coleman et al., 2013).
9. Develops relationship with patient/family/caregiver (*n* = 5; 33.3%). This involved establishing a relationship of trust (Minnesota Health Literacy Partnership, 2016), and making shared decisions with patients and family members (Kaper et al., 2018).
10. Conducts interdisciplinary collaboration (*n* = 2; 13.3%). The last subcategory included involving more than one HCP in the patient's care and establishing health education plans in cooperation with other HCPs (Chang, Chen, Wu, et al., 2017).

**Table A:**
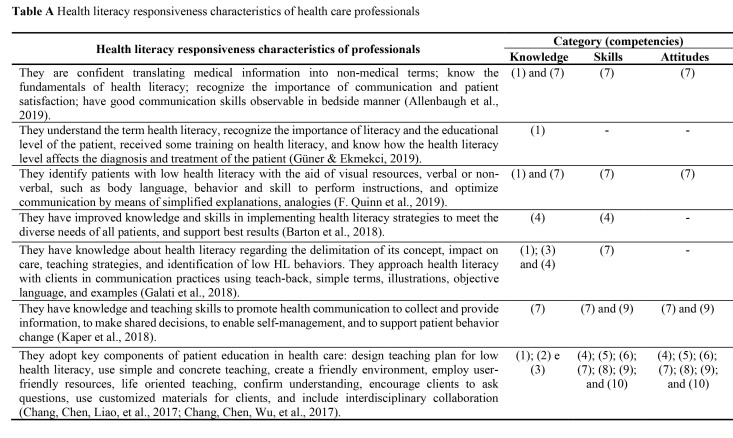

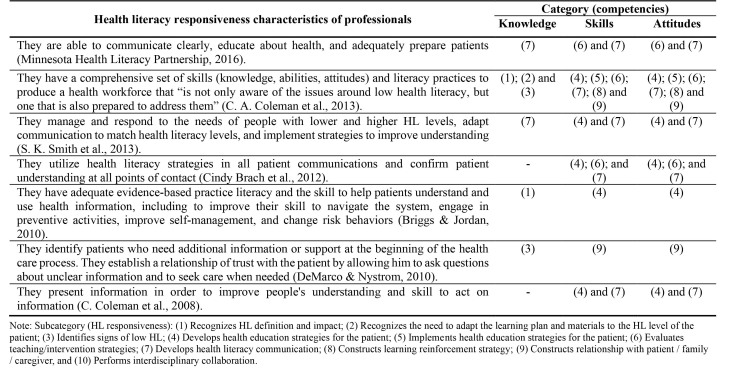
Health literacy responsiveness characteristics of health care professionals

**Health literacy responsiveness characteristics of professionals**	**Category (competencies)**		
	**Knowledge**	**Skills**	**Attitudes**
They are confident translating medical information into non-medical terms; know the fundamentals of health literacy; recognize the importance of communication and patient satisfaction; have good communication skills observable in bedside manner (Allenbaugh et al., 2019).	(1) and (7)	(7)	(7)
They understand the term health literacy, recognize the importance of literacy and the educational level of the patient, received some training on health literacy, and know how the health literacy level affects the diagnosis and treatment of the patient (Güner & Ekmekci, 2019).	(1)	-	-
They identify patients with low health literacy with the aid of visual resources, verbal or non-verbal, such as body language, behavior and skill to perform instructions, and optimize communication by means of simplified explanations, analogies (F. Quinn et al., 2019).	(1) and (7)	(7)	(7)
They have improved knowledge and skills in implementing health literacy strategies to meet the diverse needs of all patients, and support best results (Barton et al., 2018).	(4)	(4)	-
They have knowledge about health literacy regarding the delimitation of its concept, impact on care, teaching strategies, and identification of low HL behaviors. They approach health literacy with clients in communication practices using teach-back, simple terms, illustrations, objective language, and examples (Galati et al., 2018).	(1); (3) and (4)	(7)	-
They have knowledge and teaching skills to promote health communication to collect and provide information, to make shared decisions, to enable self-management, and to support patient behavior change (Kaper et al., 2018).	(7)	(7) and (9)	(7) and (9)
They adopt key components of patient education in health care: design teaching plan for low health literacy, use simple and concrete teaching, create a friendly environment, employ user-friendly resources, life oriented teaching, confirm understanding, encourage clients to ask questions, use customized materials for clients, and include interdisciplinary collaboration (Chang, Chen, Liao, et al., 2017; Chang, Chen, Wu, et al., 2017).	(1); (2) e (3)	(4); (5); (6); (7); (8); (9); and (10)	(4); (5); (6); (7); (8); (9); and (10)
They are able to communicate clearly, educate about health, and adequately prepare patients (Minnesota Health Literacy Partnership, 2016).	(7)	(6) and (7)	(6) and (7)
They have a comprehensive set of skills (knowledge, abilities, attitudes) and literacy practices to produce a health workforce that “is not only aware of the issues around low health literacy, but one that is also prepared to address them” (C. A. Coleman et al., 2013).	(1); (2) and (3)	(4); (5); (6); (7); (8) and (9)	(4); (5); (6); (7); (8) and (9)
They manage and respond to the needs of people with lower and higher HL levels, adapt communication to match health literacy levels, and implement strategies to improve understanding (S. K. Smith et al., 2013).	(7)	(4) and (7)	(4) and (7)
They utilize health literacy strategies in all patient communications and confirm patient understanding at all points of contact (Cindy Brach et al., 2012).	-	(4); (6); and (7)	(4); (6); and (7)
They have adequate evidence-based practice literacy and the skill to help patients understand and use health information, including to improve their skill to navigate the system, engage in preventive activities, improve self-management, and change risk behaviors (Briggs & Jordan, 2010).	(1)	(4)	(4)
They identify patients who need additional information or support at the beginning of the health care process. They establish a relationship of trust with the patient by allowing him to ask questions about unclear information and to seek care when needed (DeMarco & Nystrom, 2010).	(3)	(9)	(9)
They present information in order to improve people's understanding and skill to act on information (C. Coleman et al., 2008).	-	(4) and (7)	(4) and (7)

Note: Subcategory (HL responsiveness): (1) Recognizes HL definition and impact; (2) Recognizes the need to adapt the learning plan and materials to the HL level of the patient; (3) Identifies signs of low HL; (4) Develops health education strategies for the patient; (5) Implements health education strategies for the patient; (6) Evaluates teaching/intervention strategies; (7) Develops health literacy communication; (8) Constructs learning reinforcement strategy; (9) Constructs relationship with patient / family / caregiver, and (10) Performs interdisciplinary collaboration.

We identified the following categories of HCPs' responsiveness to HL: knowledge (*n* = 13; 86.7%), skills (*n* = 14; 93.3%), and attitudes (*n* = 12; 80%) **(Table A)**. HCPs' responsiveness to HL was characterized as those who know the definition and implications of HL for the well-being of individuals, and can develop, adapt, implement, and evaluate health teaching strategies according to the patient's HL.

### Interventions to Develop the HL Responsiveness of HCPs to the Patients

The interventions identified in the review totaled 19 strategies, divided into 5 subcategories **(Table B)**:

1. Expositive (*n* = 18; 94.7%). Includes interventions that used instructional methods, such as lecture (Ogrodnick et al., 2020), study session with presentation (Trujillo & Figler, 2015) and lectures (Niemi et al., 2018).
2. Interactive (*n* = 9; 47.4%). Includes activities using group dynamics for learning, with focus groups (Kaper et al., 2018) and team-based teaching clinics (Marion et al., 2018).
3. Practice with educational materials (*n* = 2; 10.5%). This included development (Shaikh et al., 2018) and evaluation of educational materials (Hadden, 2015).
4. Standardized patient or simulation (*n* = 8; 42.1%). This included the use of simulated care with a standardized patient (Marion et al., 2018), scenarios where participants played roles (Mackert et al., 2011), and simulation centers (Ogrodnick et al., 2020).
5. Practices with actual patients (*n* = 4; 21.1%). This involved actions in the community (Niemi et al., 2018), assessment of HL practices in professional settings (Trujillo & Figler, 2015), and recording of meetings with patients (Green et al., 2014).

**Table B:**
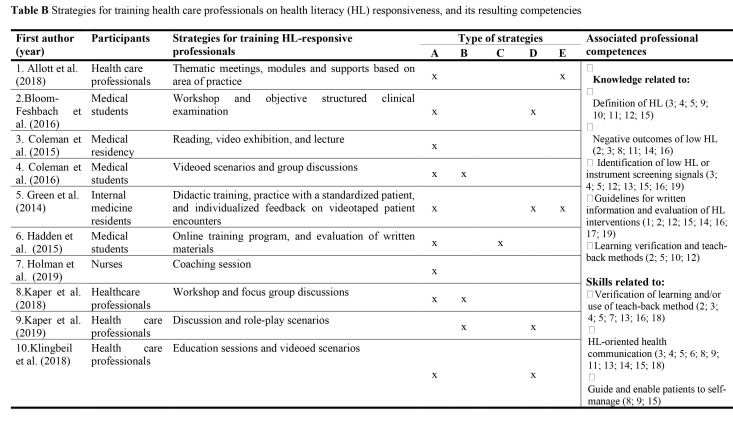

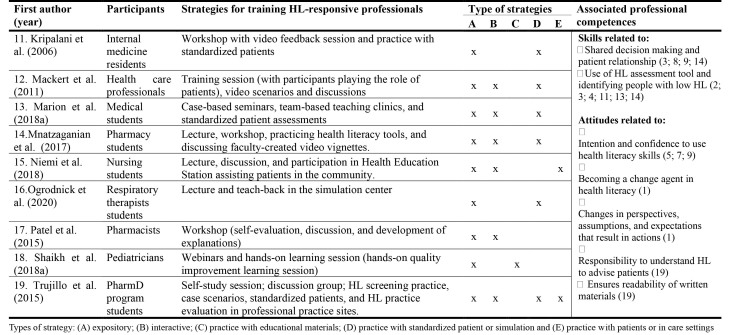
Strategies for training health care professionals on health literacy (HL) responsiveness, and its resulting competencies

**First author (year)**	**Participants**	**Strategies for training HL-responsive professionals**	**Type of strategies**					**Associated professional competences**
			**A**	**B**	**C**	**D**	**E**	
1. Allott et al. (2018)	Health care professionals	Thematic meetings, modules and supports based on area of practice	x				x	**Knowledge related to:** □ Definition of HL (3; 4; 5; 9; 10; 11; 12; 15) □ Negative outcomes of low HL (2; 3; 8; 11; 14; 16) □ Identification of low HL or instrument screening signals (3; 4; 5; 12; 13; 15; 16; 19) □ Guidelines for written information and evaluation of HL interventions (1; 2; 12; 15; 14; 16; 17; 19) □ Learning verification and teach-back methods (2; 5; 10; 12) **Skills related to:** □ Verification of learning and/or use of teach-back method (2; 3; 4; 5; 7; 13; 16; 18) □ HL-oriented health communication (3; 4; 5; 6; 8; 9; 11; 13; 14; 15; 18) □ Guide and enable patients to self-manage (8; 9; 15)
2.Bloom-Feshbach et al. (2016)	Medical students	Workshop and objective structured clinical examination	x			x		
3. Coleman et al. (2015)	Medical residency	Reading, video exhibition, and lecture	x					
4. Coleman et al. (2016)	Medical students	Videoed scenarios and group discussions	x	x				
5. Green et al. (2014)	Internal medicine residents	Didactic training, practice with a standardized patient, and individualized feedback on videotaped patient encounters	x			x	x	
6. Hadden et al. (2015)	Medical students	Online training program, and evaluation of written materials	x		x			
7. Holman et al. (2019)	Nurses	Coaching session	x					
8.Kaper et al. (2018)	Healthcare professionals	Workshop and focus group discussions	x	x				
9.Kaper et al. (2019)	Health care professionals	Discussion and role-play scenarios		x		x		
10.Klingbeil et al. (2018)	Health care professionals	Education sessions and videoed scenarios	x			x		
11. Kripalani et al. (2006)	Internal medicine residents	Workshop with video feedback session and practice with standardized patients	x			x		**Skills related to:** □ Shared decision making and patient relationship (3; 8; 9; 14) □ Use of HL assessment tool and identifying people with low HL (2; 3; 4; 11; 13; 14) **Attitudes related to:** □ Intention and confidence to use health literacy skills (5; 7; 9) □ Becoming a change agent in health literacy (1) □ Changes in perspectives, assumptions, and expectations that result in actions (1) □ Responsibility to understand HL to advise patients (19) □ Ensures readability of written materials (19)
12. Mackert et al. (2011)	Health care professionals	Training session (with participants playing the role of patients), video scenarios and discussions	x	x		x		
13. Marion et al. (2018a)	Medical students	Case-based seminars, team-based teaching clinics, and standardized patient assessments	x	x		x		
14.Mnatzaganian et al. (2017)	Pharmacy students	Lecture, workshop, practicing health literacy tools, and discussing faculty-created video vignettes.	x	x		x		
15. Niemi et al. (2018)	Nursing students	Lecture, discussion, and participation in Health Education Station assisting patients in the community.	x	x			x	
16.Ogrodnick et al. (2020)	Respiratory therapists students	Lecture and teach-back in the simulation center	x			x		
17. Patel et al. (2015)	Pharmacists	Workshop (self-evaluation, discussion, and development of explanations)	x	x				
18. Shaikh et al. (2018a)	Pediatricians	Webinars and hands-on learning session (hands-on quality improvement learning session)	x		x			
19. Trujillo et al. (2015)	PharmD program students	Self-study session; discussion group; HL screening practice, case scenarios, standardized patients, and HL practice evaluation in professional practice sites.	x	x		x	x	

Types of strategy: (A) expository; (B) interactive; (C) practice with educational materials; (D) practice with standardized patient or simulation and (E) practice with patients or in care settings

The strategies were associated with 15 competencies for HL practice. In the subcategory of knowledge (*n* = 13), these included HL definition (*n* = 8; 42.1%), low HL signal identification or instrument screening (*n* = 8; 42.1%), and guidelines for written information and intervention evaluation (*n* = 8; 42.1%). In the subcategory of skills (*n* = 14), HL-oriented health communication was the most cited item in the articles (*n* = 11; 57.9%). In the subcategory of attitude (*n* = 5), the intention for and confidence in using HL skills was the most cited item (*n* = 3; 15.8%).

In the context of the categories, types of strategies and professional competences resulting from interventions, suggests that the use of more than one strategy for developing HL responsiveness can result in a set of knowledge, skills, and attitudes related to HL.

## Discussion

The characteristics of HL responsiveness in HCPs were established by summarizing 10 subcategories of professional skills. In addition, we analyzed five categories of training strategies for both professionals and students in the HL context, mapping the potential knowledge, skills, and attitudes related to them.

The competencies theory (Perrenoud, 2013) can be a mechanism for understanding the characteristics and development of HL responsiveness in HCPs. These professionals have mastery of the theoretical component, and develop the habit of performing skills, as well as having a professional identity focused on HL, according to the results of this study. Thus, competence can be an indicator of responsiveness.

A recent literature review showed educational activities in the classroom, simulation laboratory, and practice with patients to be elements for the development of curricula that prepare health students in the context of HL (Saunders et al., 2018). These results are consistent with our findings, which also identified other interventions, including professional qualification, in addition to student education. This study also categorized the competencies required for interventions to enable appropriate teaching strategies addressing health service needs.

From this perspective, the present study showed that education of HCPs using interactive strategies, practice with educational materials, and actual or simulated patients have more potential to add skills to the professional's learning. However, the expository or instructional component is relevant to consolidate the theoretical components among participants of the interventions (Coleman et al., 2016; Marion et al., 2018).

The constructivist teaching-learning approach explains the best adjustment of these methodologies with the development of competencies. From this perspective, learning is a product of doing, and the environment (or experiencing reality) acts as a facilitator (Piaget, 2013). For this reason, more practice sessions and use of scenarios with actual or simulated patients can promote the development of knowledge, skills, and attitudes in nursing students (Marion et al., 2018; Niemi et al., 2018; Ogrodnick et al., 2020) and professionals (Allott et al., 2018; Kaper et al., 2019; Shaikh et al., 2018).

The results showed that only one-quarter (*n* = 5; 26.3%) of 19 studies that performed interventions with professionals or students in the context of HL evaluated attitudes. This fact is probably related to the difficulty of measuring changes in attitude after the interventions. Attitude is related to knowing how to do, and wanting to do, which can result in subjective and not always observable data (Bloom et al., 1956; Perrenoud, 2013).

Moreover, knowledge and skills do not guarantee practice (attitude). Health literacy needs to be part of values, principles, and manner of interpersonal relationship of HCPs. In this sense, an individual, organizational, and systemic approach can contribute to favorable attitudes of the HCPs regarding HL and enable them to meet the HL demands of the population (Allott et al., 2018).

At the individual level, the positive role of understanding HL as a personal responsibility is important (Patel, 2015), as well as having leaders who support these actions, and being part of a team that advocates HL principles in care practices (Allott et al., 2018). The second level comprises organizational aspects, which includes having HL principles in organizational strategic plans and human resource management. The systemic level includes having HL incorporated into national, state, and regional health plans (Allott et al., 2018).

## Limitations and Implications for Future Research

This study has some limitations. First, this review was limited to research presented in English, Spanish, or Portuguese. There may have been search terms that would have captured more concepts. This project focuses on the health care professional but there are other dimensions of organizational improvement for which the provider may need to be responsive to decrease health literacy barriers. In addition, we describe interventions for development of HL—responsiveness in HCPs without analyzing their level of evidence.

## Conclusions

This review mapped 14 characteristics, classified 10 sub-categories of responsiveness, and proposed characteristics of HCP responsiveness to health literacy. The professional responsive to the patient's health literacy was characterized as one who knows the definition and implications of health literacy for patient well-being, and can develop, adapt, implement, and evaluate health education strategies. We identified 19 strategies for preparing professionals responsive to the patient's health literacy, classified as expository, interactive, practice with educative materials, practice with standardized patients or simulation, and practice with actual patients. The mapped characteristics and interventions are precursors for the development of curricula and professional education programs, as well as the development and use of measures to evaluate the health workforce.
